# Editorial: Insights in neurocognitive aging and behavior: 2021

**DOI:** 10.3389/fnagi.2023.1147618

**Published:** 2023-02-07

**Authors:** Kristy A. Nielson, Ian M. McDonough, Anja Soldan

**Affiliations:** ^1^Director, Aging, Imaging and Memory (AIM) Laboratory, Department of Psychology, Marquette University, Milwaukee, WI, United States; ^2^Alabama Research Institute on Aging, Department of Psychology, The University of Alabama, Tuscaloosa, AL, United States; ^3^Cognitive Neuroscience Division, Department of Neurology, Johns Hopkins University School of Medicine, Baltimore, MD, United States

**Keywords:** brain aging, Alzheimer's disease, neuroimaging, biomarkers, cognitive reserve, cognitive aging theories, lifestyle activities, exercise

In recent years, exceptional scientific achievements have led to major advancements in the fast-growing field of neurocognitive aging and behavior. In this inaugural collection, *Insights in Neurocognitive Aging and Behavior: 2021*, we sought to highlight the latest advancements and challenges for the current state of knowledge and future directions in aging neuroscience in the neurocognitive arena. Here we outline the contributions and implications for future research of the 15 papers in this topic collection across four important research areas: (1) novel approaches to identifying and tracking brain aging and impending cognitive decline; (2) neurocognitive markers of risks for Alzheimer's disease (AD) and its progression; (3) lifestyle contributions to cognitive aging and AD; and (4) the status and future of neurocognitive and brain aging theory.

## Insights in novel indices of neurocognitive aging

Thirty years of advances in neuroimaging that allow us to visualize brain structure and function *in vivo* have transformed how we think about the aging brain (Risacher and Saykin, 2019). This topic collection addresses this, but also several newer approaches that have promise to further revolutionize our understanding of the aging brain. Huang et al. examined the cognitive implications of carotid artery stenosis (CAS) and associated leukoaraiosis in middle to older age, as it is a prevalent condition in aging. Covarying multiple other relevant factors, they found direct effects of left CAS on most cognitive tests, except visual memory and construction, which were instead influenced primarily by right CAS. Their findings point to possible new directions in early detection and intervention of cognitive decline and to novel insights into biological asymmetries in brain functioning. Similarly innovative, Wang et al. examined retinal thickness and microvasculature as a rapid, non-invasive, and accessible screening approach to detect impending cognitive decline in middle and older age. Retinal structure and microvasculature were associated with cognition (e.g., processing speed) and hippocampal volume. These studies offer the potential for new insights into early mechanisms and detection of risk for cognitive decline, with approaches that might also better reach underserved populations.

Two studies in the collection examined metabolic factors as indices of cognitive functioning. Ayerdem et al. studied erythropoietin (EPO), a hormone that stimulates red blood cell production, in a large sample of community-dwelling middle-aged and older adults. Higher EPO levels were associated with better complex cognition (executive functioning), suggesting it may offer neurocognitive protection. Higher serum ferritin, the major iron storage protein, was contrastingly associated with poorer episodic memory. Thus, basic blood-based markers may be able to predict cognitive decline, thereby providing new directions for studying the biological pathways of cognitive aging. Relatedly, Li et al. examined motoric cognitive risk syndrome (MCR; 10% prevalence in older adults), a “pre-dementia syndrome” characterized by slow but unassisted gait and subjective cognitive complaints without dementia. Metabolomic and lipidomic profile analysis revealed distinct metabolic subtypes associated with MCR, as distinguished from the profiles in subjects with cognitive impairment. Their findings shed new light on metabolic contributions to subjective cognitive complaints and risk for cognitive decline, as well as on the mechanisms of MCR.

## Insights in neurocognitive markers of AD risk and progression

AD, the most common form of dementia, has no cure or highly effective treatment to stop its progression (Alzheimer's Association., 2022). Three studies in this collection addressed new directions in detection and diagnosis of AD. The first study examined autosomal dominant AD (ADAD; 1% of AD cases, Bekris et al., 2010). ADAD has long asymptomatic and only mildly symptomatic phases (Ryman et al., 2014), thereby allowing study of early biomarkers and pathophysiological changes. Qiu reviewed resting and task-related functional magnetic resonance imaging (fMRI) activity and connectivity studies in ADAD, reporting abnormalities in key hubs in medial temporal lobe, striatum, posterior cingulate, and frontal cortices. Similar patterns were evident in sporadic AD, with both AD types distinguishable from those in typical aging. Inter-network connectivity was also greatest prior to symptom onset, with decreasing connectivity as symptoms progress and age of typical onset approaches. Their findings, if applied in large, multi-center studies, might provide a model for predicting AD onset and progression. Dong et al. examined functional connectivity data using machine learning to predict neuropsychological performance in AD and mild cognitive impairment (MCI) compared with healthy controls. Cognitive impairment was effectively predicted by three networks—central executive, sensorimotor, and default mode. Notably, multiple rather than single networks were implicated in each cognitive domain, suggesting that assessing the extent of multi-network changes might be key to predicting MCI and AD. Lastly, Murakami and Lacayo used the 2022 Reactome pathway knowledgebase and GeneAnalytics to identify and update the current listing of genetic and disease hallmarks for AD. Their work added five new AD biological hallmarks to existing hallmarks. They further showed that AD genes were associated not just with AD but also with >20 diverse age-related diseases and comorbidities, suggesting that various modifiable lifestyle factors are the crucial targets for AD prevention and treatment (Smith et al., 2013).

## Insights in lifestyle contributions to cognitive aging and AD

Modifiable lifestyle behaviors (e.g., exercise, diet, social engagement, etc.) have widespread associations with brain functioning that are protective of brain and cognition in aging (McDonough et al., 2020; Soldan et al., 2021; Won et al., 2021); they have been proposed to reduce the risk of dementia by 40% (Livingston et al., 2020). Three papers in this topic collection addressed the influence of lifestyle factors in cognitive aging. Festini's mini-review outlined evidence of the benefits of living a busy and socially, physically, and mentally engaged lifestyle. She also highlighted that the mechanisms underlying these effects are unknown and that since “busyness” is neither inherently beneficial or detrimental on its own, better study designs are needed to decipher the complex inter-relationships amongst busyness, stress, and cognition. Gillespie et al. examined MRI, genetic, and environmental factors on predicted brain age in a large longitudinal sample of male twins. They found early and genetic influences were highly correlated and attributable to a single, common factor that was stable across age and thus, were unlikely to greatly contribute to brain aging. In contrast, individual factors such as relationships, diet, drugs, stressors, and lifestyle activities, were more likely to impact brain age, with negative life events, particularly in interpersonal relationships, most negatively associated with brain age. Finally, Gust et al. illuminated a critical young-old cohort difference that might explain age-related functional connectivity differences: fitness. Baseline data from a 16-week exercise intervention study comparing young and older adults showed that although average connectivity did not differ between the age groups, region-to-region connectivity was weaker within the fronto-parietal and default-mode networks in older adults. After accounting for fitness, age differences were attenuated, suggesting that age-related fitness loss may drive regional interconnectivity changes.

## Insights and new avenues in neurocognitive and brain aging theory

At least four major, sometimes opposing, patterns of brain aging are often discussed in the literature: loss of neural distinctiveness, neural inefficiency, neural compensation, and brain maintenance (Dennis and Gutchess, 2020). Three papers in this collection addressed neurocognitive aging theories, highlighting their role in future scientific progress. A review by McDonough et al. noted that many papers report brain aging patterns that have not replicated, or that are potentially mutually exclusive, thereby conflicting with one another. They concluded that no current theory is specific or robust enough to delineate when such patterns will or will not occur in a given sample. They highlighted the need for pre-registration of theories, larger and diverse samples, and explicit testing of theory with specific, directional hypotheses. Similarly, a bibliometric analysis by Othman et al. concluded that no one existing theory fully explains the variability in cognitive aging patterns. They argued that the literature is increasingly scattered, with too few authors making multiple contributions to the field, and for the urgent need to integrate findings with modern theory across multiple major arenas, from individual level processing to risk factors and population studies.

Two review papers in this collection are good examples of the limitations of modern neurocognitive aging theories and their applications. Cansino conducted a systematic review of successful episodic memory retrieval in aging, as measured by functional MRI (fMRI) connectivity. Surprisingly, only twenty studies were eligible for the review, but unsurprisingly, the majority used the hippocampus as the primary seed, which directed the focus of the analysis. Older adults had decreased hippocampal connectivity with the recollection network (e.g., parahippocampal gyrus, posterior cingulate cortex), but increased connectivity with various distant, task-relevant regions, relative to young adults. She speculated that the latter was compensatory, albeit at heightened cost, to overcome reduced function in the recollection network. Relatedly, Long et al. examined aging across 278 fMRI studies of inhibitory control, a fundamental aspect of executive functioning. Their meta-analysis suggested a core network underlies both cognitive- and response-inhibition, as separate components (e.g., middle cingulate, supplementary motor area, inferior frontal gyrus, inferior parietal lobule, and insula), while some regions had more component-specific roles, such as superior parietal lobule in cognitive inhibition and right inferior frontal gyrus, bilateral insula, and angular gyrus in response inhibition. Moreover, left inferior frontal gyrus, bilateral insula and left superior parietal lobule activation diverged in older adults, being greater during cognitive inhibition but reduced during response inhibition. Thus, with complex conflict processing, flexible regional recruitment appeared limited in older adults. Yet, in both these studies, interpretations were mostly speculative due to studies lacking direct theoretical comparisons and to overlaps of theoretical predictions.

Kremen et al. reviewed the literature on another theoretically important arena of cognitive aging research, cognitive reserve, brain reserve, and resilience. These are hypothetical constructs describing individual differences in the ability of the brain or mind to resist damage and degeneration (Pettigrew and Soldan, 2019; Stern et al., 2020). Due to inconsistencies in the usage of these terms and resulting empirical confounds, the authors proposed new definitions to specify and distinguish reserve, maintenance, and resilience as parallel constructs. They organized these definitions around cognitive reserve, cognitive resilience, and the maintenance of cognitive reserve on the one hand, and brain reserve, brain resilience, and brain maintenance on the other hand. This new framework may guide future research into individual differences in cognitive and brain aging trajectories and the various lifestyle and genetic factors that contribute to them. We note that there is also need for these constructs to better integrate with contemporary brain and cognitive aging theories.

## Conclusion

Despite the advances in neuroimaging and the proposal of multiple neurocognitive aging theories, the studies in this inaugural collection highlight that a new frontier is coming. New research is on the cusp of better integrating existing knowledge that will allow researchers to better predict cognitive decline and accompanying brain changes that occur with aging and in age-related diseases on an individual basis, or at least for subsets of individuals (Albert et al., 2018; Paitel and Nielson, 2021). Some of this new research stems from a better understanding of how peripheral organs that interact with the brain can be leveraged. Other approaches highlight the growing need to incorporate adults in middle-age rather than assuming the underlying mechanisms related to cognitive decline and brain alterations start in older age. Doing so pushes the boundary of early detection and moves toward life course approaches to our understanding of neurocognitive aging (McDonough et al., 2019).

## Author contributions

KN wrote the first draft of the manuscript. All authors contributed to manuscript additions and revisions, and read and approved the submitted version.

## References

[B1] Albert M. Zhu Y. Moghekar A. Mori S. Miller M. I. Soldan A.. (2018). Predicting progression from normal cognition to mild cognitive impairment for individuals at 5 years. Brain. 141, 877–887. 10.1093/brain/awx365 29365053 PMC5837651

[B2] Alzheimer's Association. (2022). 2022 Alzheimer's disease facts and figures. Alzheimers Dement. 18, 700–789. 10.1002/alz.12638 35289055

[B3] Bekris L. M. Yu C. E. Bird T. D. Tsuang D. W. (2010). Genetics of Alzheimer disease. J. Geriatr. Psychiat. Neurol. 23, 213–227. 10.1177/0891988710383571 21045163 PMC3044597

[B4] Dennis N. A. Gutchess A. (2020). Overview of models of cognitive aging, in The Cambridge Handbook of Cognitive Aging: A Life Course Perspective, eds. Thomas A. K. Gutchess A. (Cambridge: Cambridge University Press) 5–31. 10.1017/9781108552684.002

[B5] Livingston G. Huntley J. Sommerlad A. Ames D. Ballard C. Banerjee S.. (2020). Dementia prevention, intervention, and care: 2020 report of the Lancet Commission. Lancet. 396, 413–446. 10.1016/S0140-6736(20)30367-6 32738937 PMC7392084

[B6] McDonough I. M. Festini S. B. Wood M. M. (2020). Risk for Alzheimer's disease: A review of long-term episodic memory encoding and retrieval fMRI studies. Ageing Res. Rev. 62, 101133. 10.1016/j.arr.2020.101133 32717407

[B7] McDonough I. M. Letang S. K. Stinson E. A. (2019). Dementia risk elevates brain activity during memory retrieval: a functional MRI analysis of middle aged and older Adults. J. Alzheimers Dis. 70, 1005–1023. 10.3233/JAD-190035 31306118

[B8] Paitel E. R. Nielson K. A. (2021). Temporal dynamics of event-related potentials during inhibitory control characterize age-related neural compensation. Symmetry. 13, 2323. 10.3390/sym13122323 35923222 PMC9345327

[B9] Pettigrew C. Soldan A. (2019). Defining cognitive reserve and implications for cognitive aging. Curr. Neurol. Neurosci. Rep. 19, 1. 10.1007/s11910-019-0917-z 30627880 PMC7812665

[B10] Risacher S. L. Saykin A. J. (2019). Neuroimaging in aging and neurologic diseases. Handb. Clin. Neurol. 167, 191–227. 10.1016/B978-0-12-804766-8.00012-1 31753134 PMC9006168

[B11] Ryman D. C. Acosta-Baena N. Aisen P. S. Bird T. Danek A. Fox N. C.. (2014). Symptom onset in autosomal dominant Alzheimer disease: a systematic review and meta-analysis. Neurology. 83, 253–260. 10.1212/WNL.0000000000000596 24928124 PMC4117367

[B12] Smith J. C. Nielson K. A. Woodard J. L. Seidenberg M. Rao S. M. (2013). Physical activity and brain function in older adults at increased risk for Alzheimer's disease. Brain Sci. 3, 54–83. 10.3390/brainsci3010054 24961307 PMC4061823

[B13] Soldan A. Pettigrew C. Zhu Y. Wang M. C. Bilgel M. Hou X.. (2021). Association of lifestyle activities with functional brain connectivity and relationship to cognitive decline among older adults. Cereb. Cortex. 31, 5637–5651. 10.1093/cercor/bhab187 34184058 PMC8568002

[B14] Stern Y. Arenaza-Urquijo E. M. Bartrés-Faz D. Belleville S. Cantilon M. Chetelat G.. (2020). Whitepaper: Defining and investigating cognitive reserve, brain reserve, and brain maintenance. Alzheimers Dement. 16, 1305–1311. 10.1016/j.jalz.2018.07.219 30222945 PMC6417987

[B15] Won J. Callow D. D. Pena G. S. Jordan L. S. Arnold-Nedimala N. A. Nielson K. A.. (2021). Hippocampal functional connectivity and memory performance after exercise intervention in older adults with Mild Cognitive Impairment. J. Alzheimers Dis. 82, 1015–1031. 10.3233/JAD-210051 34151792 PMC8461699

